# Exopolysaccharide Producing *Bifidobacterium animalis* subsp. *lactis* Strains Modify the Intestinal Microbiota and the Plasmatic Cytokine Levels of BALB/c Mice According to the Type of Polymer Synthesized

**DOI:** 10.3389/fmicb.2020.601233

**Published:** 2020-11-26

**Authors:** Carlos Sabater, Natalia Molinero-García, Nuria Castro-Bravo, Patricia Diez-Echave, Laura Hidalgo-García, Susana Delgado, Borja Sánchez, Julio Gálvez, Abelardo Margolles, Patricia Ruas-Madiedo

**Affiliations:** ^1^Department of Microbiology and Biochemistry of Dairy Products, Instituto de Productos Lácteos de Asturias – Consejo Superior de Investigaciones Científicas (IPLA-CSIC), Villaviciosa, Spain; ^2^Microhealth Group, Instituto de Investigación Sanitaria del Principado de Asturias (ISPA), Oviedo, Spain; ^3^CIBER-EHD, Department of Pharmacology, Center for Biomedical Research (CIBM), University of Granada, Granada, Spain; ^4^Instituto de Investigación Biosanitaria de Granada (ibs.GRANADA), Granada, Spain

**Keywords:** exopolysaccharide, bifidobacteria, microbiota, mice, cytokine, immune response

## Abstract

Bacteria-host interactions are mediated by different microbial associated molecular patterns which are most often surface structures such as, among others, exopolysaccharides (EPSs). In this work, the capability of two isogenic EPS-producing *Bifidobacterium animalis* subsp. *lactis* strains to modulate the gut microbiota of healthy mice, was assessed. Each strain produces a different type of polymer; the ropy strain S89L synthesized a rhamnose-rich, high-molecular weight EPS in highest abundance than the non-ropy DMS10140 one. BALB/c mice were orally fed for 10 days with milk-bifidobacterial suspensions and followed afterward for 7 post-intervention days (wash-out period). The colonic content of mice was collected in several sampling points to perform a metataxonomic analysis. In addition, the influence of specific microbial clades, apparently stimulated by the ropy and non-ropy strains, on mouse plasmatic cytokine levels was investigated through hierarchical association testing. Analysis of 16S rRNA gene sequences showed that the abundance of *Firmicutes* phylum significantly increased 7 days after cessing the treatment with both strains. The relative abundance of *Alloprevotella* genus also rose, but after shorter post-treatment times (3 days for both DMS10140 and S89L strains). Some bacterial clades were specifically modulated by one or another strain. As such, the non-ropy DMS10140 strain exerted a significant influence on *Intestinomonas* genus, which increased after 4 post-administration days. On the other hand, feeding with the ropy strain S89L led to an increase in sequences of *Faecalibaculum* genus at 4 post-treatment days, while the abundance of *Erysipelotrichaceae* and *Lactobacillaceae* families increased for prolonged times. Association testing revealed that several lactobacilli and bifidobacterial significantly stimulated by ropy S89L strain were positively associated with the levels of certain cytokines, including IL-5 and IL-27. These results highlight relevant changes in mice gut microbiota produced after administration of the ropy S89L strain that were associated to a potential immune modulation effect.

## Introduction

*Bifidobacterium* is part of the human intestinal microbiota being one of the most abundant genera in this ecosystem, mainly in infants. Furthermore, some bifidobacterial strains are considered to have probiotic properties and, thus, they are commonly used in commercial probiotic products. Some of their health properties could be related to the presence of specific surface molecules, such as exopolysaccharides (EPS). EPS are carbohydrate polymers synthesized by some bacteria that can be totally liberated to the extracellular milieu, or can remain loosely attached to the bacterial surface. EPS play an important role in the intestinal ecosystem by mediating bacterial-host interactions, modulating the immune system response, and also acting as a fermentable carbon source by other members of the microbiota (Castro-Bravo et al., 2018). Besides, these polymers are of great importance for the producing bacteria due to their protective role against adverse conditions, thus allowing their persistence in the gut for a longer time (Fanning et al., 2012). EPS structures contain different monomers, mainly D-glucose, D-galactose, and L-rhamnose, although others can be found such as *N*-acetyl glucosamine, D-glucose, D-ribose, or fucose. Some of these bacterial EPS can be used by the human gut microbiota leading to a high production of short chain fatty acids (Salazar et al., 2016; Liu et al., 2019). It has been demonstrated that some EPS-producing bifidobacteria can modulate the intestinal microbiota diversity and function (by modifying the profile of released metabolites), as determined *in vitro* by means of pH-controlled fecal batch fermentations or *in vivo* using animal experimental studies (Salazar et al., 2016; Yan et al., 2019). Microbiota modulation in inflammatory processes can be of great importance. Recent studies describe the evolution of gut microbiome during inflammatory bowel disease and detect relevant relationships between certain taxa and serum levels of antibodies through hierarchical association models (Lloyd-Price et al., 2019). Among the biological activities of EPS described, also the structure-immunity relationships have gained great attention (Hidalgo-Cantabrana et al., 2012; Xu et al., 2019). In fact, two recent studies carried out with an EPS-producing *Bifidobacterium longum* strain in a murine model of DSS-induced colitis showed that the strain was able to alleviate the inflammatory symptoms through the microbiota modulation and the maintenance of the mucosal barrier (Yan et al., 2019, 2020).

Several studies dealing with EPS producing *Bifidobacterium animalis* subsp. *lactis* strains, specifically those having a “ropy” phenotype due to the production of a rhamnose-rich high molecular weight (HMW)-EPS, have been carried out in our research group. It has been demonstrated that strains producing this ropy EPS can *in vitro* elicit different immune responses when co-incubated with PBMC (peripheral blood mononuclear cells) from humans (López et al., 2012) or with GALT (gut associated lymphoid tissue) isolated from rats (Hidalgo-Cantabrana et al., 2014), being also able to ameliorate inflammatory symptoms in a DSS-induced colitis mice model (Hidalgo-Cantabrana et al., 2016). However, so far, no studies demonstrating whether the intestinal microbiota could be differentially modulated by ropy and non-ropy strains in a healthy animal model have been reported. Therefore, the aim of this study was to determine the capability of different EPS-producing *B. animalis* subsp. *lactis* strains to modify the intestinal microbiota in healthy BALB/c mice and to explain the influence of relevant clades stimulated by EPS on the production of serum cytokines in animals with a non-disturbed mucosal barrier. To achieve this, a recently developed bacterial model, based on the wild-type strain *B. animalis* subsp. *lactis* DSM10140 (non-ropy) and its ropy isogenic mutant S89L that produces the HMW-EPS in higher abundance, was selected. Mutant S89L was obtained using gene replacement techniques to substitute the gene *balat_1410* (responsible for the determination of the EPS chain length) of DSM10140 for the mutated one with a single nucleotide change (Castro-Bravo et al., 2017). Therefore, differences between both strains on microbial and immune modulation capabilities could be only attributed to a single gene which determines the chain size of the polymer. Then, the main difference between both strains is that S89L presents in its surface a bigger amount of the HMW-EPS than its parental DSM10114 strain.

## Materials and Methods

### Bacterial Growth Conditions

The DSM101410 and S89L strains were cultivated in MRSc [MRS (Biokar Diagnostics) supplemented with 0.25% L-cysteine-HCl (Sigma-Chemical Co.)] at 37°C, for 24 h, in a jar under anaerobic conditions (Anaerocult A, Merck). Cultures were washed with PBS and resuspended in heat-treated 11% skimmed milk (BD Difco, Thermo Fisher Scientific Inc.). Bifidobacterial suspensions in milk, containing on average 8.9 ± 0.4 Log CFU/ml, of each strain were daily prepared to be administered (dose of 100 μl) to the mice by means of a gastric tube.

### Experimental Design

The animal experimental procedure was approved by the Ethical Committee of Laboratory Animals of the University of Granada (Spain) (Ref. No. CEEA-2010-286). Female BALB/c mice (7–9 weeks old, approximately 20 g) were obtained from Janvier Labs (St Berthevin Cedex) and kept under conventional conditions with a standard pelleted diet and sterilized water for 1 week before beginning the experiments. A total of 114 animals were randomly distributed in three groups: 48 mice receiving the non-ropy strain (DSM10140 group), 48 mice receiving the ropy strain (S89L group) and the 18 remaining mice did not receive any treatment (reference group). The weight at the beginning of the experimental procedure for each animal group was (mean ± SD): 22.28 ± 1.45 g for the control group, 22.82 ± 1.56 g for DSM10140-treated group and 22.14 ± 1.65 for g the S89L-treated group. The statistical analysis performed (ANOVA) showed no statistical differences in the initial weight among the three groups (*p* > 0.05). The experimental design was as follows: 10 days of intervention with daily oral administration of the milk-bifidobacteria suspensions, followed by 7 days of post-intervention without bifidobacteria intake (wash-out period). No variations in behavior or health status were observed in the three groups of mice during the experimentation period. In different sampling points (5 and 10 days of intervention, and 1, 3, 4, and 7 post-intervention days), 8 mice from each bifidobacterial group, and 3 mice from the reference group (thus, 18 control animals in total), were sacrificed in order to collect the colonic content and the blood serum. For that, each mouse was anesthetized with an overdose of halothane and blood was extracted from the heart using heparinized tubes. After that, animals were sacrificed by cervical dislocation and the gut was excised to collect its content. Samples were stored at −80°C after their collection.

### Cytokine Analysis

The ProcartaPlex Multiplex Immunoassay for mouse (Thermo Fisher Scientific Inc.) was used to quantify the levels of different cytokines (IL-1β, IL-2, IL-4, IL-5, IL-6, IL-9, IL-10, IL-12p70, IL-13, IL-17a, IL-18, IL-22, IL-23, IL-27, GM-CSF, IFN-γ, and TNF-α) by means of the FACS Canto II flow cytometer (BD Biosciences). The detection limits (pg/mL) for those detected in our samples were: 2.21 ± 0.36 (IL-5), 5.48 ± 0.72 (IL-6), 15.08 ± 0.62 (IL-9), 2.27 ± 0.01 (IL-10), 1.65 ± 0.36 (IL-17a), 12.30 ± 0.80 (IL-23), 2.68 ± 0.01 (IL-27), and 1.12 ± 0.19 (IFN-γ).

### 16S rRNA and 16S-23S Internal Transcribed Spacer (ITS) Gene Sequencing

Total DNA was isolated from the colonic content of the 114 samples using the QiaEz DNA-extraction protocol previously optimized in our research group (Milani et al., 2013), consisting in a mechanical cell disruption step, followed by enzymatic lysis and combined with an extraction with the QIAamp Stool DNA kit (Qiagen). Using the primers Probio_Uni and Probio_Rev (Milani et al., 2013), the V3 region of the 16S rRNA gene was amplified. The 16S-23S Internal Transcribed Spacers (ITS) were amplified from extracted DNA using the specific primer pair ProbioBif-ITS_Fw and ProbioBif-ITS_Rev, which targets the variable region between the 16S rRNA and 23S rRNA gene sequences (Milani et al., 2014). Sequencing was performed using an Illumina MiSeq machine at GenProbio S.R.L. (Parma, Italy). Sequence reads were filtered and the resulting ones were processed using a personalized script of QIIME software (Caporaso et al., 2010) matched by pair-ends. Quality control filtering was performed, keeping sequences with a mean sequence quality score >20 and a length between 140 and 400 bp.

### Data Analysis

Shapiro–Wilk’s Normality test (*p* < 0.05) and Levene’s test (*p* < 0.05) to determine the homogeneity of variances were calculated for all data generated. Statistically significant differences between samples were calculated through Kruskal–Wallis and Mann–Whitney statistical tests for non-parametric independent samples followed by False Discovery Rate (FDR) *post hoc* using Benjamini–Hochberg method with a value of 0.25. All statistical analyses were computed on R v3.5.0 and Mothur software.

In order to calculate diversity measures, the 16S rRNA reads were clustered in Operational Taxonomic Units (OTUs) defined at ≥99% sequence homology by meanings of UCLUST software (Edgar, 2010). All reads were classified to the lowest possible taxonomic rank using the QIIME and the reference database SILVA (Quast et al., 2013). Similarity of the microbial communities between the samples was calculated by UniFrac method (Lozupone and Knight, 2005). Phyloseq (McMurdie and Holmes, 2013) and Microbiome (Lahti and Shetty, 2017) packages were also used in the analysis of sequencing data. After assignment of reads to phylum, family, genus and species levels, two differential analyses were performed: Metastast algorithm of Mothur software, and DESeq2 differential abundance testing for sequencing data (Love et al., 2014). To determine statistical differences in taxonomic data according to the strain, treatment time or both *p*_*adj*_ lower than 0.05 and log2FoldChange greater than 1.5 were considered.

Relevant taxonomic clades were associated to cytokine plasmatic levels through hierarchical all-against-all association testing (HAIIA)^1^ (Lloyd-Price et al., 2019) considering *q*-values and a Bonferroni False Discovery Rate of 0.25. In addition, a graphical correlation network between taxa and cytokines was computed using ccrepe (Schwager et al., 2019) and qgraph (Epskamp et al., 2012) packages.

## Results

### Influence of Ropy and Non-ropy Strains on Cytokine Levels

**FIGURE 1:**
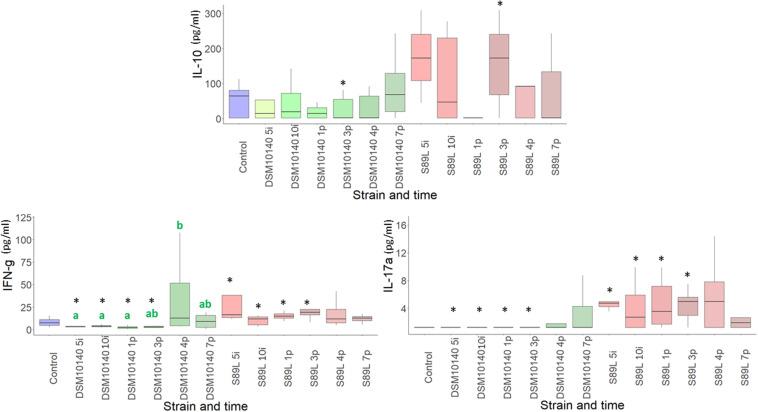
Box plot (median, interquartile range, minimum and maximum values) of the level (pg/ml) of IL-10, IFN-γ, IL-17A quantified in the blood serum of control mice group, as wells as in the groups fed with the non-ropy DSM10140 or with the ropy S89L *Bifidobacterium animalis* subsp. *lactis* strains at different sampling points (5i and 10i days of intervention, and 1p, 3p, 4p, and 7p days post-intervention). Within each strain-treatment group, samples (days) that do not share a common letter are statistically different (*p* < 0.05). Within each treatment day, the statistical differences between the DSM10140 and S89L groups are marked with an asterisk (*p < 0.05). For information about other cytokines, see Supplementary Table 1 in the Supplementary Material.

The administration of non-ropy DSM10140 and ropy S89L *B. animalis* subsp. *lactis* strains to BALB/c mice exerted a relevant influence on the levels of some serum cytokines. Statistically significant differences in their production according to the strain selected (ropy or non-ropy) as well as the treatment time (5 and 10 days of intervention, and 1, 3, 4, and 7 post-intervention days) were found (Figure 1, the statistical analysis is shown in Supplementary Table 1). Regarding the anti-inflammatory IL-10, although no statistical differences were detected (with the exception of sample 3p), levels were higher after S89L administration compared to DMS1040 strain. On the other hand, significantly lower levels of IFN-γ and IL-17a were found in mice treated with DSM10140 than in treated with S89L during the intervention period and until the first 3 post-intervention days. However, no differences were detected between both mice groups when the ratios IL-10/INF-γ or IL-10/IL-17a were calculated, although a tendency to have higher values (i.e., anti-inflammatory profile) for the S89L treated group was detected, probably due to the highest production of IL-10 (Supplementary Table 1). The release of other cytokines, such as IL-9, IL-23, and IL-27, was also enhanced in S89L treatments (Supplementary Table 1), although after 4 post-treatment days no differences between both mice groups were detected. In both mice groups, similar concentrations of other quantifiable cytokines, such as IL-5 and IL-6, were detected, but the last one tend to be released in lower concentrations in mice fed with the ropy S89L strain. The levels of the others analyzed (IL-1β, IL-2, IL-4, IL-10, IL-12p70, IL-13, IL-18, IL-22, GM-CSF, and TNF-α) were below the limit of detection of the method used (Supplementary Table 1).

After a principal component analysis (PCA) of this data, no clear patterns could be inferred in the general cytokine profile obtained, since the PCA explains a low percentage (below 50%) of variance (Supplementary Figure 1). These results could be related with the high inter-individual variability found among mice.

### Influence of Ropy and Non-ropy Strains on Gut Microbiota

**FIGURE 2:**
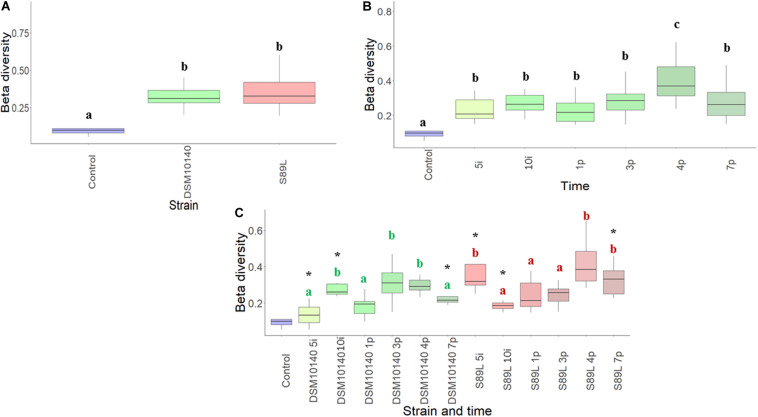
Beta-diversity (Bray–Curtis metrics) of the relative abundance of colonic microbiota samples from mice grouped according to the EPS-strain used for feeding **(A)**, the intervention time **(B)** and a combination of both EPS-strain and sampling (days) points **(C)**. Different letters indicate statistical differences among the different EPS-strain groups **(A)** or sampling days **(B)**; in the combination of both factors **(C)**, within each EPS-strain group samples (days) those that do not share a common letter are statistically different (*p* < 0.05), whereas within each treatment day, the statistical differences between the DSM10140 and S89L groups are marked with an asterisk (*p < 0.05).

The influence of the ropy and non-ropy EPS-producing strains on mice gut microbiota composition was studied. First, the alpha diversity measuring variability of species within a sample was calculated using different indices (Shannon, Simpson, and Inverse Simpson) which provide complementary information, reflecting different patterns in the core microbiota. These indexes showed a greater dispersion for mice fed with non-ropy DSM10140 and ropy S89L strains in comparison to the control group, which was more accentuated during the post-intervention period, especially in S89L group (Supplementary Figure 2). The global Chao1 index, an estimation of the number of species represented by only one individual in the sample, was 90.3, ranging from 89.6 to 93.0 in DSM10140 group and from 82.0 to 91.4 in S89L-treated mice. On the other hand, the beta-diversity (Bray–Curtis distance) measuring differences in composition among samples, revealed that DSM10140 and S89L groups had a more diverse microbiota than the control group (Figure 2A), especially at the end of the post-intervention period (Figure 2B), achieving a higher number of species at the fourth day of post-intervention for the ropy S89L treatment (Figure 2C). These results suggest that the administration of non-ropy DSM10140 and ropy S89L strains stimulate a significant number of species that are present in low abundances in the control group. In addition, the ropy EPS seems to exert a stronger effect on gut microbiota that takes place mainly at the final days of the post-treatment period.

**FIGURE 3:**
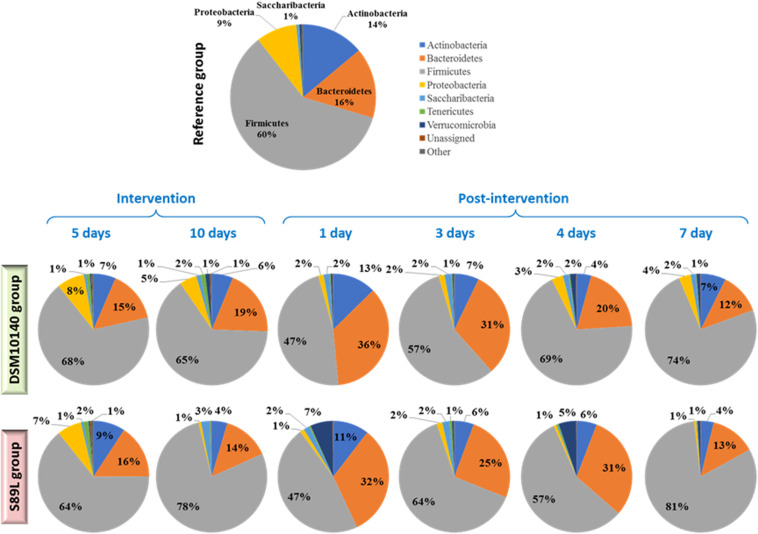
Relative abundance of the main (≥1%) bacterial phyla detected in the colonic content of different BALB/c mice groups (*n* ≥ 6 per group and sampling day). The reference group was constituted by non-treated mice of the same litter; treatment groups were fed for 10 days with a daily dose of 8.9 ± 0.4 Log CFU/ml, suspended in skimmed milk, of the EPS-producing strains *B. animalis* subsp. *lactis* DSM10140 (parental, non-ropy strain) or S89L (mutant, ropy strain producing a HMW-EPS). Colonic content samples were obtained from mice sacrificed at two points during the intervention period (5 and 10 days) and at four points post-intervention (without administration of bifidobacteria: 1, 3, 4, and 7 days). For statistical analysis see Supplementary Table S2 in Supplementary Material.

The relative abundance of the main (≥1%) bacterial phyla detected in the colon content of the different BALB/c mice groups is represented in Figure 3 (the complete statistical analysis is provided in Supplementary Table 2). In general, the evolution of abundances along the intervention and post-intervention period was similar in both bifidobacterial-treated mice groups. Specifically, percentages remained without changes (*p* > 0.05) between the two sampling points (5i and 10i) of the intervention period and they resemble that of the reference group. However, at 1 and 3 post-intervention days statistical differences (*p* < 0.05) for some phyla were detected with respect to the intervention period and also with respect to longer times of the post-intervention. In fact, it seems that the more time elapsed since the end of intervention (4 and 7 days, post-intervention), the more resembles the profile of the microbiota phyla to the initial state and to the reference (non-treated) group. Thus, the greatest changes were detected after 1 and 3 days of cessation of bifidobacterial intake; an increase in Actinobacteria and Bacteroidetes abundance, to detriment of the Firmicutes phylum, was found. This indicates that the biggest influence of our EPS-producing bifidobacteria on the colonic microbiota occurred during the wash-out period, after finishing the oral administration of both strains, suggesting that this intervention with our EPS-producing bifidobacteria had a delayed effect on microbiota dynamics in agreement with diversity analyses previously described. The other (most abundant) phylum presenting variations along the intervention period was Proteobacteria (Figure 3 and Supplementary Table 2); a reduction in the relative abundance was observed from the beginning of the intervention period to the end of the experimental procedure in both bifidobacterial-treatment groups, but this effect only remained in the group S89L fed with the ropy-EPS bifidobacteria. Indeed after 4 and 7 days post-intervention the group fed with the strain DSM10140 showed higher (*p* < 0.05) relative abundance of this phylum than the S89L-fed group (Supplementary Table 2). In both cases, the levels of Proteobacteria were significantly lower than in the control group. To corroborate these statistically significant differences, DESeq2 differential abundance testing was applied to all phyla determined (Supplementary Figure 3). It was also found that Tenericutes were significantly higher in mice fed with ropy and non-ropy strains compared to control groups (Supplementary Figure 3A). Proteobacteria abundance decreased during post-intervention period when combined data from both treatments (Supplementary Figure 3B) in agreement with the results presented in Figure 3. Moreover, significant differences were observed for Firmicutes considering both the selected strain and intervention time separately (Supplementary Figure 3C); higher abundances at the end of the intervention (10i point) in S89L group were observed. However, in both groups of mice, a relevant increase in the relative abundance of this phylum was achieved after 7 post-treatment days in agreement with the non-parametric statistical tests presented in Figure 3.

**FIGURE 4:**
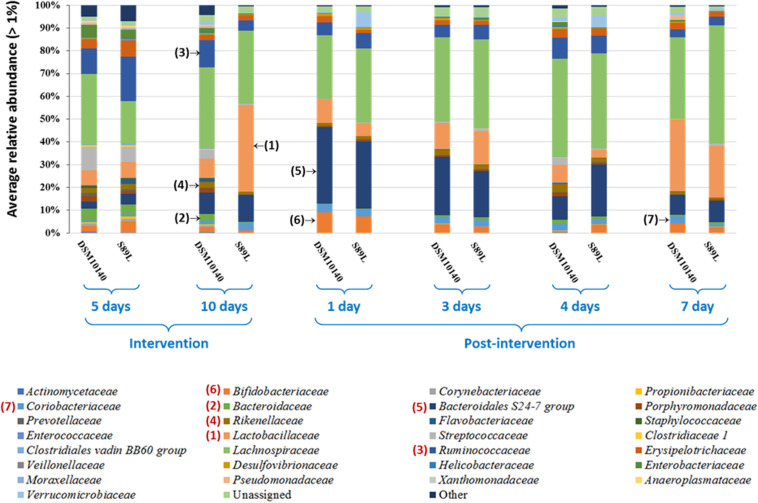
Mean (*n* ≥ 6 mice per group and sampling day) of the bacterial families, having a relative abundance percentage higher than 1%, present in the colon content of mice treated with the EPS-producing strains *B. animalis* subsp. *lactis* DSM10140 or S89L. The treatment procedure is described in the legend of Figure 3. For statistical analysis see Supplementary Table S3 in Supplementary Material.

Regarding family level analysis, differences of relative abundances along the experimental points (Figure 4), as well as between both treatment mice groups at a given point (Supplementary Table 3) were observed. Among the most abundant (>2%) families, the noticeable change at the end of the intervention period (10i) was the significant (*p* < 0.05) increase in *Lactobacillaceae*, which was also corroborated by DESeq2 differential abundance test (Supplementary Figure 4A). A decrease in *Bacteroidaceae* and *Ruminococcaceae* (*Clostridia* class) in the S89L group was also denoted (Figure 4 and Supplementary Table 3) then, concomitantly, there was an increase of Firmicutes/Bacteroidetes ratio at this 10i point (Supplementary Figure 5). Finally, the S89L treatment group also showed a significant decrease of *Rikenellaceae* (Bacteroidetes phylum) with respect to the DSM10140-fed group (Supplementary Table 3). During the post-intervention (wash-out) period, a remarkable increase of *Bacteroidales* S24-7 family was detected at day 1 by both conventional statistical tests (Figure 4) and DESeq2 differential abundance testing (Supplementary Figure 4B) in both groups of mice, which tended to decline afterward with a concomitant long-term increase of *Lactobacillaceae*. Curiously, in the first post-intervention day the *Bifidobacteriae* family increased its relative abundance in both groups of mice (Figure 4), and the percentages fluctuated along wash-out period detecting significant differences between DSM10140 and S89L groups at the 4th post-intervention day (Supplementary Table 3). At this day, there were also significant (*p* < 0.05) differences on the relative abundance of *Desulfovibrionaceae* family between treatment groups, being lower the percentage in S89L-treated mice. It should be noted that advanced DESeq2 differential abundance test also detected significant changes in minor families present in mice microbiota (>0.1%). For example, *Lachnospiraceae* increased during post-intervention period as well as *Erysipelotrichaceae*, which achieved higher abundances at the fourth day after ropy S89L treatment. Similarly, *Coriobacteriaceae* increased during the first days of post-intervention with both strains and decreased at the seventh day probably indicating a partial loss of the modulatory activity (Supplementary Figure 4B). In general, *Verrucomicrobiaceae* showed higher abundances in mice administered with S89L, regardless time (Supplementary Figure 4A).

**FIGURE 5:**
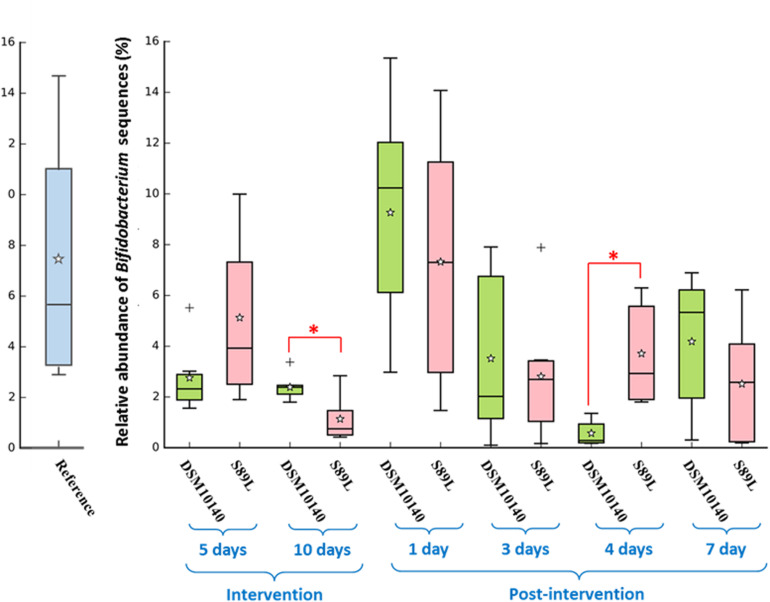
Relative abundance (%) of sequences corresponding to *Bifidobacterium* spp. in the colonic content of mice treated with the EPS-producing strains *B. animalis* subsp. *lactis* DSM10140 or S89L, and non-treated reference group. The treatment procedure is described in the legend of Figure 3. For each combination treatment day/strain, the “box and whiskers” plot represents median, interquartile range and minimum and maximum values, calculated from de sequences obtained for at least 6 mice; the average is represented with the white star. Within each treatment day, the non-parametric Mann–Whitney test for 2-independent samples was used to assess differences between both strain (*p < 0.05). The IBM SPSS Statistic vs25 package was used for the non-parametric statistical analysis.

Taxonomic analysis of genera present in samples (relative abundance > 0.1%) using DESeq2 differential abundance test revealed a general increase in *Ruminococcaceae* UCG-014 genus compared to the control group (Supplementary Figure 6A). In addition, a stimulation of *Ruminococcus gnavus* group during post-intervention was observed, which was more accentuated in S89L treatment (Supplementary Figure 6B) although no differences were found among the three groups of mice (Supplementary Figure 6A). An increase in the abundance of this bacterium has been associated with pro-inflammatory states, such as Crohn disease (Henke et al., 2019); however, as we have indicated in previous sentences, the slight increase in the relative abundance of *R. ganvus* did not correlate with a pro-inflammatory state in or experimental model. In addition, S89L administration led to a high abundance of *Eubacterium fissicatena* group and *Faecalibaculum* after 3 and 4 post-treatment days, although this modulatory activity decreased at longer times. Similarly, *Alloprevotella* and *Intestinimonas* achieved higher abundances after 2–3 post-intervention days with both strains (Supplementary Figure 6B). Given that this intervention study was carried out with two strains of *Bifidobacterium*, this genus was analyzed in more detail. Figure 5 shows the “box and whisker” plot representing the relative abundance of the sequences identified as bifidobacteria. Intriguingly, during the intervention procedure a reduction in the relative abundance of *Bifidobacterium* was observed in both bifidobacterial-treated groups with respect the levels found in the reference (non-intervention) mice group. In fact, this is coincident with the lower proportion of Actinobacteria phylum observed in the two treated mice groups in comparison to the reference one (Figure 3). It seems that this was the phylum reducing its relative abundance in higher extent during the intervention period and *Bifidobacterium* genus might account for this behavior. Indeed, the recovering of Actinobacteria phylum and *Bifidobacteriaceae* family, denoted at the first post-intervention day, also match with the increase of this genus in both treatment groups (Figure 5 and Supplementary Figure 7). Besides, the statistical differences (Supplementary Table 3) observed in the family at the 10th day of intervention, and the 4th post-intervention day between the DSM10140 and S89L groups are also coincident with the tendency observed for *Bifidobacterium* spp.

**FIGURE 6:**
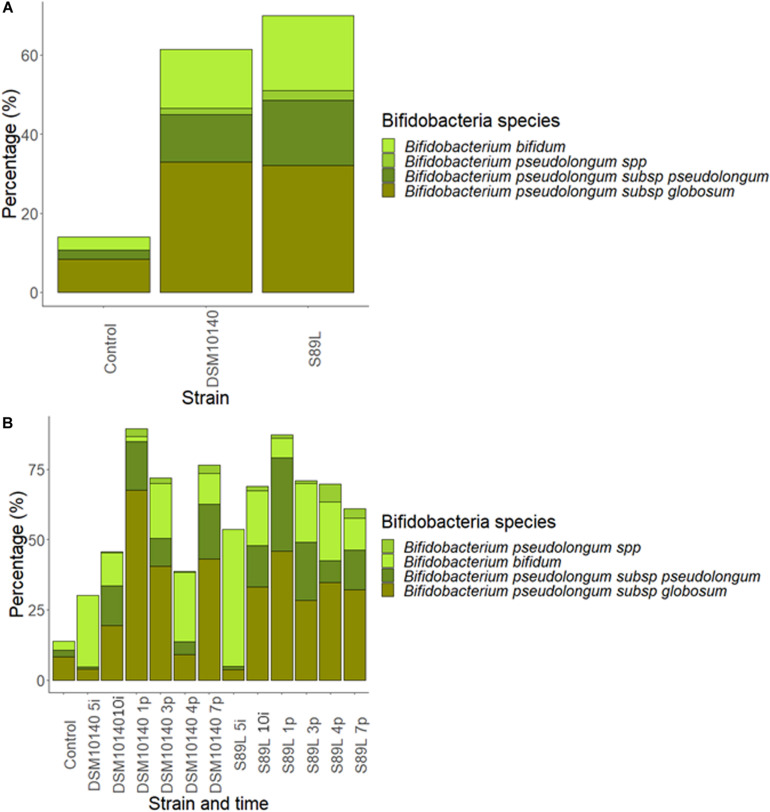
Abundance percentages of statistically significant different *Bifidobacterium* species after Internal Transcribed Spacer (ITS) sequencing according to the EPS-strain selected **(A)** and both EPS-strain and intervention time **(B)**.

To complete taxonomic characterization, a tentative species-level analysis was also performed. For this purpose, statistically relevant lactobacilli and bifidobacteria (>0.1%) determined by DESeq2 differential abundance test was carried out with the 16S rRNA gene sequences. Abundances of *B. animalis* were increased in both treatments with respect to the control group (Supplementary Figure 8A), but no statistical differences were found. Curiously, a great increase in *Lactobacillus reuteri* populations was achieved at the end of post-intervention period with both strains (Supplementary Figure 8B), corroborating the delayed effect of the microbiota modulatory activity previously observed for other clades. Further, the ITS regions were sequenced to study the species belonging to *Bifidobacterium* genus (Figure 6). Administration of both strains enhanced *B. bifidum* growth compared to the control group, regardless intervention time (Figure 6A). On the other hand, the abundances of *B. pseudolongum* subsp. *globosum* and *B. pseudolongum* subsp. *pseudolongum* showed a great abundance during post-intervention in both treatments while *B. pseudolongum* spp. growth significant increased at 4 days of post-treatment in S89L group (Figure 6B). It was previously reported that animals fed with EPS-producing bifidobacteria are able to increase the populations of other bifidobacterial species (Salazar et al., 2011), which could be explained by the use of the polymers as fermentable substrates for other microbiota inhabitants, including bifidobacteria (Salazar et al., 2016).

### Associations Between Serum Cytokine Levels and Gut Microbiota

**FIGURE 7:**
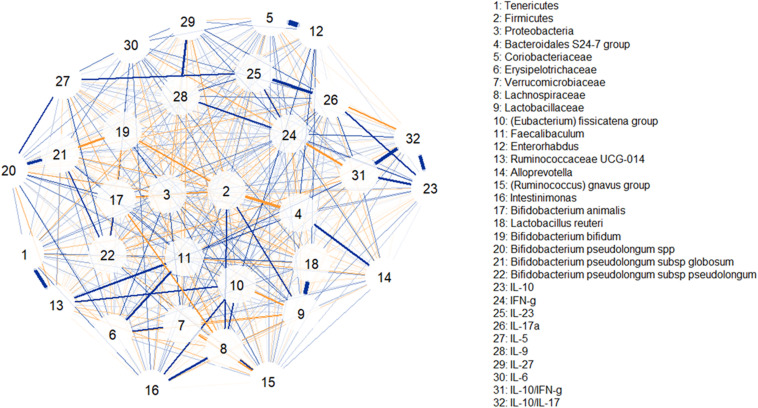
Correlation network illustrating positive and negative associations between taxonomic clades, serum levels of cytokines and inflammatory factors. Blue lines indicate positive associations while saffron lines suggest negative associations. Line thickness is in proportion to magnitude.

**FIGURE 8:**
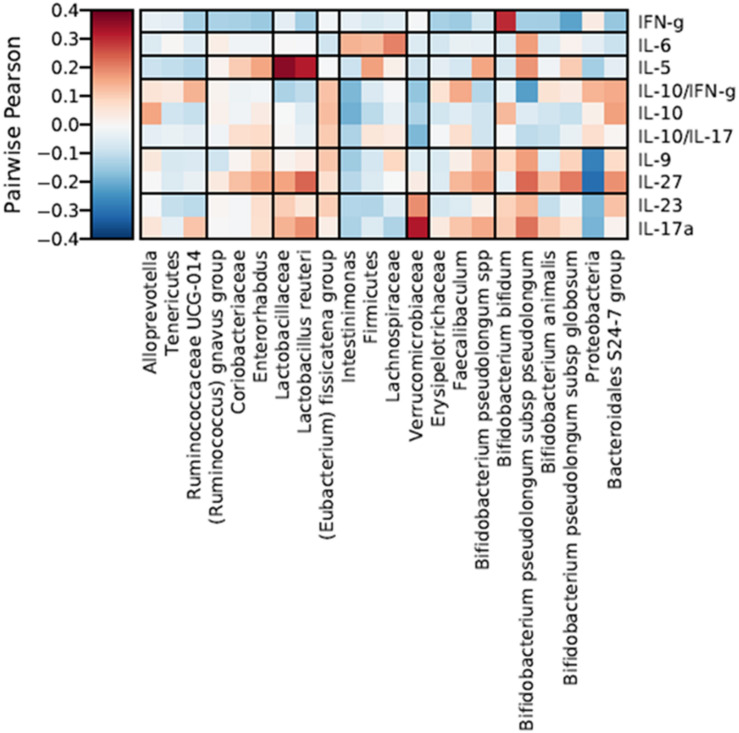
Hierarchical all-against-all association testing (HAllA) describing the effect of specific taxonomic clades on serum levels of cytokines and inflammatory factors, considering Pairwise Pearson correlation coefficients adjusted by False Discovery Rate (FDR < 0.25). Red and blue cells indicate positive and negative correlations, respectively. Color intensity is in proportion to magnitude.

To better understand the potential biological effect of the administration of S89L ropy strain in mice, serum cytokine markers and microbial taxonomic data were integrated through correlation networks (Figure 7) and hierarchical all-against-all association testing (HAIIA; Figure 8). Positive and negative associations between taxonomic data and cytokine profiles were first represented as a correlation network (Figure 7). As expected, *Lactobacillaceae* family was positive associated to *Lactobacillus* species while different species of bifidobacteria positively associated to each other. In this sense, positive relationships between *Coriobacteriaceae* family and *Enterorhabdus* genus are due to the shared class *Coriobacteria*. Positive relationship between *Tenericutes* and *Rumicococcaceae* UCG-014 as well as between *Lachnospiraceae* family and *Intestinimonas* were observed. As expected, abundances of Firmicutes and *Bacteroidales* S-24 group were correlated in a negative way. With regard to cytokine profiles, in general, they showed positive correlations to each other. The HAIIA analysis (Figure 8) revealed that *Lactobacillaceae* family and specifically *L. reuteri* were associated to high levels of IL-5. Similarly, populations of *B. pseudolongum* subspecies and *L. reuteri* were associated to higher serum levels of IL-27. It is worth remember that the S89L strain (ropy- EPS) stimulated the growth of these microorganisms, exerting a delayed effect that took place during the post-intervention time. *Verrucomicrobiaceae* family slightly contributed to the release of IL-17a cytokine and the decrease of IL-10/IL-17a ratio. It has also been observed that the IFN-γ was positively associated to *B. bifidum*, and negatively associated to *B. pseudolongum* subsp. *globosum*, indicating a different modulatory pattern. With regard to other clades, Proteobacteria, reduced during post-intervention, is associated to a lower release of interleukins IL-9 and IL-27.

## Discussion

A differential modulatory effect of two isogenic EPS-producing *B. animalis* subsp. *lactis* strains on both gut microbiota and cytokine production in a healthy mice model has been found. Potential relationships between the different microbial clades determined were elucidated through correlation networks. As expected, different lactobacilli and bifidobacteria were positively associated with other members of *Lactobacillaceae* and *Bifidobacteriaceae*. Other interestingly relationships suggested include positive correlations between Tenericutes and *Rumicococcaceae* UCG-014 (*Firmicutes*) in agreement with Zhang and Gao (2017) which reported that bacterial genomes in the phyla Tenericutes and Firmicutes are notably positively correlated. *Lachnospiraceae* family and *Intestinimonas* genus were positively correlated as both clades involving butyrate producers (Bui et al., 2016). In contrast, Firmicutes and *Bacteroidales* S-24 group were negatively associated, and antagonic relationships between these two clades have been already reported in humanized mice (Clarke et al., 2012). Indeed, the Firmicutes/Bacteroidetes (F/B) ratio was shown to be an indicator of human gut microbiota status and a variation in this value has been related to different pathological states, such as autoimmune diseases, metabolic syndrome or obesity, among others (Hevia et al., 2014). The tendency is different according to the disease; for example, in obesity the intestinal microbiota dysbiosis was correlated with an increase in the F/B ratio where more abundance of Firmicutes is observed in different types of experiments (Castaner et al., 2018), whereas the contrary was found in systemic lupus erythematous disease (Hevia et al., 2014). In our study, this ratio decreased in both bifidobacterial mice groups in the first stages of post-intervention (Supplementary Figure 5); this agrees with a previous study in which *Bifidobacterium pseudocatenulatum* (strain CECT7765) was administered to mice, although in a model of obesity (Moya-Pérez et al., 2015). However, in our case this effect reverted in further post-intervention days tending to reach the initial ratio, or even increase it, at the last day of the experimental follow up. This behavior could be explained by the fact the orally administered bacteria are not able to permanently colonize the gut ecosystem which will tend to reach the initial microbial homeostasis after cessation the intake of both EPS-producing *B. animalis* subsp. *lactis* strains. Regarding Proteobacteria phylum, which was the decreased in mice at the end of both experimental treatments, is normally present at low levels in a healthy human gut but, under certain conditions, the opportunistic members of the phylum can overgrow, such as in the case of inflammatory diseases or aging. It has been proved a positive correlation between opportunistic enterobacteria and some pro-inflammatory markers in elders (Biagi et al., 2010). Contrarily, treatment with specific *Bifidobacterium* strains can down-regulate the postoperative pro-inflammatory response of patients undergoing colorectal resection (Mizuta et al., 2016). It has been reported that *Rikenellaceae* (Bacteroidetes phylum) family, which decreased in ropy S89L treatment group, seems to be over-represented in genetic and diet-induced obese mice (Kim et al., 2012); thus, it could be interesting to explore the potential application of ropy EPS-producing strains to reduce the levels of *Rikenellaceae* in an obese model. The S24-7 family of the *Bacteroidales* order, which was reduced at prolonged post-intervention times, is a prominent component of the murine gut microbiota, and seems to be present as well within the human intestinal community (Ormerod et al., 2016). This LPS-producing Gram-negative group might be directly involved in the mild-inflammation states related to some physiopathological processes, such as obesity (Kang et al., 2017) or aging (Van Beek et al., 2018). Administration of ropy S89L strain led to a lower accumulation of intestinal sulfate reducing bacteria (SRB), like *Desulfovibrionaceae*, that are directly related to IBD (inflammatory bowel disease) development (Kushkevych et al., 2018). On the other hand, a significant difference at the last sampling point of our experimental procedure (7th post-intervention day) in the family *Coriobacteriaceae* was detected, showing also a reduction in S89L-fed mice group. These bacteria are normal inhabitants of the gut, where they carry out relevant functions such as the conversion of bile salts and steroids as well as the activation of dietary polyphenols. However, their increased occurrence has been associated with a range of pathologies; as an example, this taxon is over-represented in colorectal cancer-associated microbiomes and this is why some of their members could be considered as pathobionts (Tjalsma et al., 2012). The variations detected between the two ropy and non-ropy treatment groups in the relative abundance of several taxa indicate that the presence of different amounts the HMW-EPS between both strains modulated the mice microbiota in a different way. In general, the relative abundance of several bacterial groups prevalent in different immune-related disorders was under-represented in mice fed with the ropy S89L strain. Nevertheless, interventions toward reducing the levels of potential pathobionts with ropy EPS-producing strains should be further explored, being an opportunity for their application as probiotics.

On the other hand, the influence of taxonomic changes induced by these EPS-producing bifidobacteria administration on serum cytokine levels was investigated. Our results agree with those obtained by Yan et al. (2019) that reported a significant decrease in IL-6 levels in mice after treatment with ropy-EPS producing strain from *B. longum*. Immune modulatory activity of EPS from *B. animalis* subsp. *lactis* had been previously reported *in vitro*, regulating the production of IL-6 and TNF-α in a dose-dependent manner in murine macrophage cell line RAW 264.7 (Liu et al., 2017). In fact, a reduction in the IL-6 levels in rats fed with wild-type ropy *B. animalis* subsp. *lactis* strains were previously demonstrated in a rat model (Salazar et al., 2014), in the same way that we have found in the current study the mutant S89L strain. Additionally, administration of EPS from *Lactobacillus fermentum* combined with *B. animalis* subsp. *lactis* led to a relevant decrease in TNF-α and increased IL-10 production in mice (Ale et al., 2019), which was also observed in our study as well as previous ones carried out with closely related ropy *B. animalis* subsp. *lactis* strains (Hidalgo-Cantabrana et al., 2014, 2015, 2016). Moreover, high doses of EPS from *B. animalis* can contribute to maintain IL-2/IL-10 ratio in mice (Xu et al., 2017) and it has been described that EPS modulates IFN-γ and IL-10 (Xu et al., 2019), a behavior also observed in our study. Interestingly, Schiavi et al. (2016) reported that surface-associated EPS from *B. longum* subsp. *longum* modulates IL-17 levels in mice, while hierarchical association testing performed in our work suggested that *B. pseudolongum* subsp. *pseudolongum* was positively associated to a higher release of this cytokine. This test also revealed that IFN-γ was associated with some bifidobacteria, like *B. bifidum*, in agreement with previous studies (Wang et al., 2019). Results presented in the current work reinforce our previous findings about the potential anti-inflammatory effect of ropy EPS from *B. animalis* subsp. *lactis* which could be mediated through the modulation of gut microbiota.

## Conclusion

In short, the fluctuations of the relative abundance of different taxa in the colonic microbiota of the treated mice observed along this experimental procedure must be directly related to the intake of *B. animalis* subsp. *lactis* strains. The lack of consistency in the evolution of microbial populations could be linked to the high inter-individual differences among mice within each group, mainly taking into account that we have analyzed different animals in each sampling point of the intervention. In spite of this, we have found significant variations and tendencies in certain microbial groups associated with the presence of different EPS in the surface the bifidobacterial strains under study. In general, the ropy S89L strain, covered in higher proportion by rhamnose-rich HMW-EPS, reduced the abundance of microbial groups that could be related with low-degree inflammatory states. In addition, we have found that the influence of specific taxa, stimulated by the ropy and non-ropy strains, on cytokine plasmatic levels investigated through hierarchical association testing also suggests an anti-inflammatory effect of the ropy S89L orally administered in mice. Altogether, results reported here could explain previous observations with ropy EPS-producing strains. Thus, the attenuation of immune response, or the induction of an anti-inflammatory profile, by ropy EPSs could be also related to a differential modulation of the intestinal microbiota with respect to that induced by non-EPS producing strains. Further studies must be undertaken to correlate the positive and negative relations between ropy EPS-producing strains and specific microbial groups, and to propose their application for restoring the microbial dysbiosis associated with specific inflammatory diseases.

## Data Availability Statement

The datasets generated for this study can be found in online repositories. The names of the repository/repositories and accession number(s) can be found in the article/Supplementary Material.

## Ethics Statement

The animal study was reviewed and approved by Ethical Committee of Laboratory Animals of the University of Granada (Spain) (Ref. No. CEEA-2010-286).

## Author Contributions

AM and PR-M were in charge of the experimental design of this work. NC-B, PD-E, and LH-G performed the animal experimentation procedure. CS and NM-G performed the data analyses. SD, BS, and JG supervised the work of the Ph.D. students. PR-M wrote the manuscript draft. All authors have revised and approved the final version.

## Conflict of Interest

The authors declare that the research was conducted in the absence of any commercial or financial relationships that could be construed as a potential conflict of interest.

## Footnotes

1 huttenhower.sph.harvard.edu/halla
