# Supplementary material for: Exopolysaccharide Producing Bifidobacterium animalis subsp. lactis Strains Modify the Intestinal Microbiota and the Plasmatic Cytokine Levels of BALB/c Mice According to the Type of Polymer Synthesized
Source: Front Microbiol. 2020 Nov 26;11:601233. doi: 10.3389/fmicb.2020.601233 (PMC7726137; doi:10.3389/fmicb.2020.601233)
Supplement: Supplementary file 1 [file Data_Sheet_1.pdf]

DATA AVAILABILITY STATEMENT

Sequencing data files are publicly available at the Sequence Read Archive (SRA) of the National Center for Biotechnology Information (NCBI) including 16S (https://www.ncbi.nlm.nih.gov/Traces/study/?acc=PRJNA670248&o=acc_s%3Aa) and 16S-23S ITS (https://www.ncbi.nlm.nih.gov/Traces/study/?acc=PRJNA670440&o=acc_s%3Aa) reads in FASTQ format.

Data were deposited under the BioProject numbers PRJNA670248 (16S) and PRJNA670440 (16S-23S ITS), SRA accession numbers SRP288087 (16S) and SRP288086 (16S-23S ITS), and BioSample accession numbers SAMN16490486 to SAMN16490592 (16S samples) and SAMN16498988 to SAMN16499094 (16S-23S ITS samples).

Supplementary Material

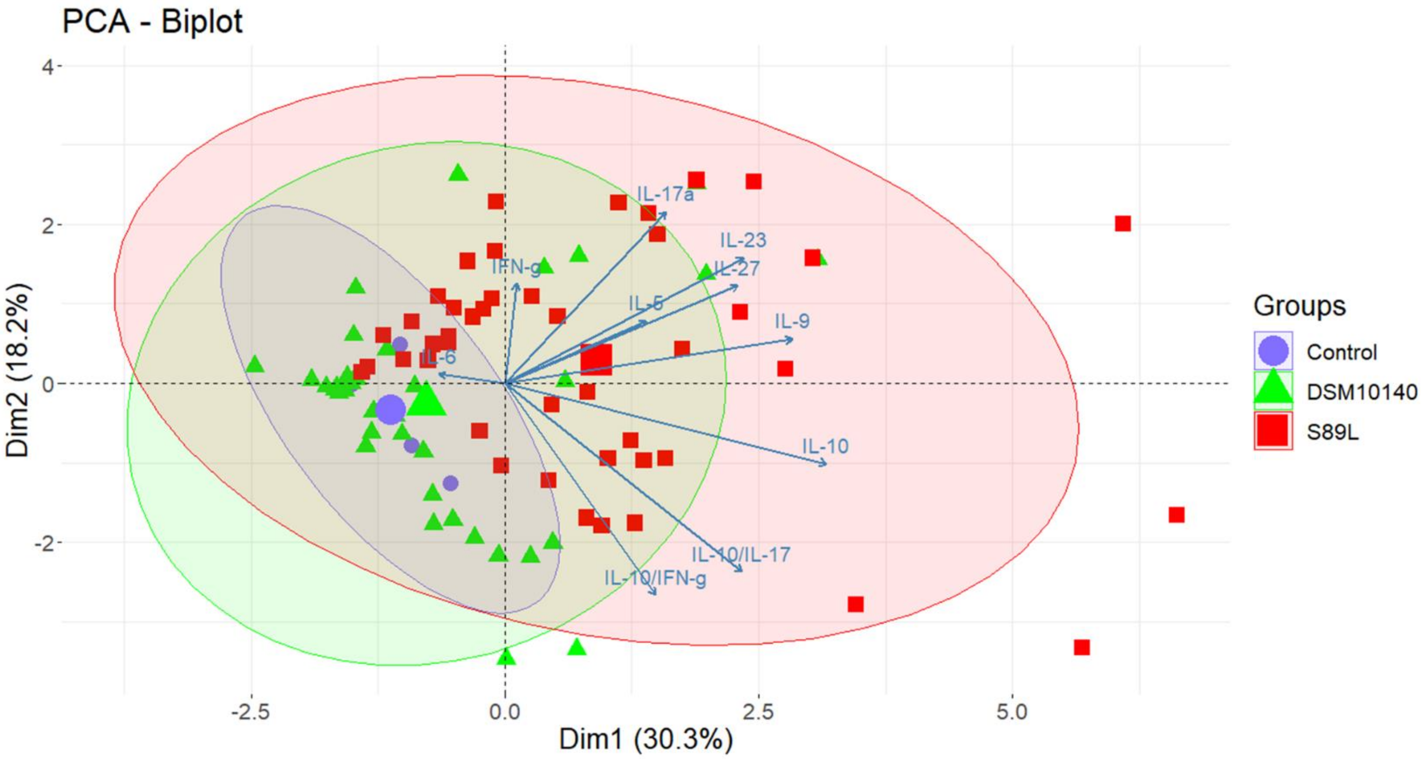

FIGURE S1 | Principal components analysis (PCA) of the quantifiable serum cytokines (see Table S1). As it can be seen, no relevant differences in the global profile of samples can be observed according to the EPS-strain selected and a low percentage of variance is explained. Dim: dimension (principal component).

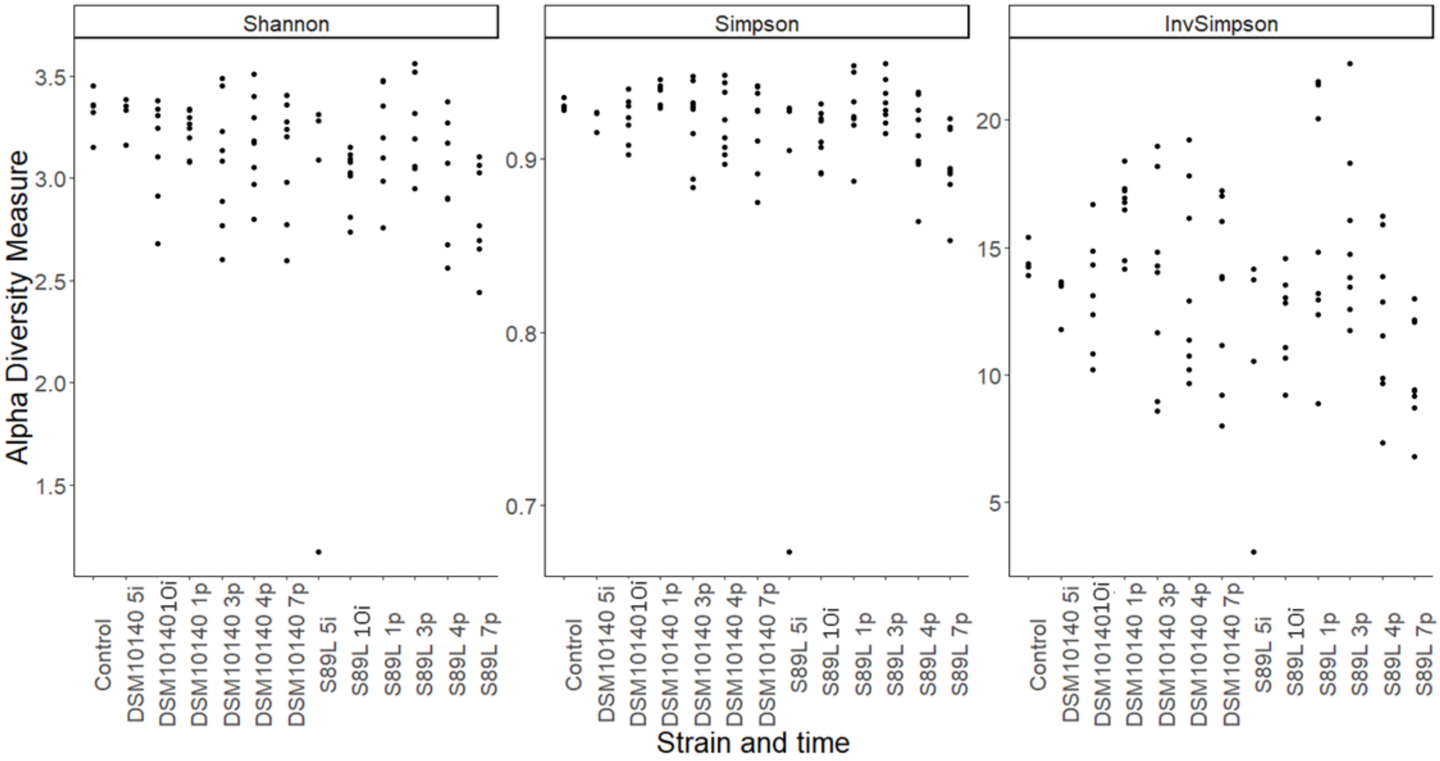

FIGURE S2 | Comparison of different alpha-diversity indicators (Shannon, Simpson and Inverse Simpson) of the relative abundance obtained after sequencing the microbiota colonic samples according to both EPS-strain and intervention time.

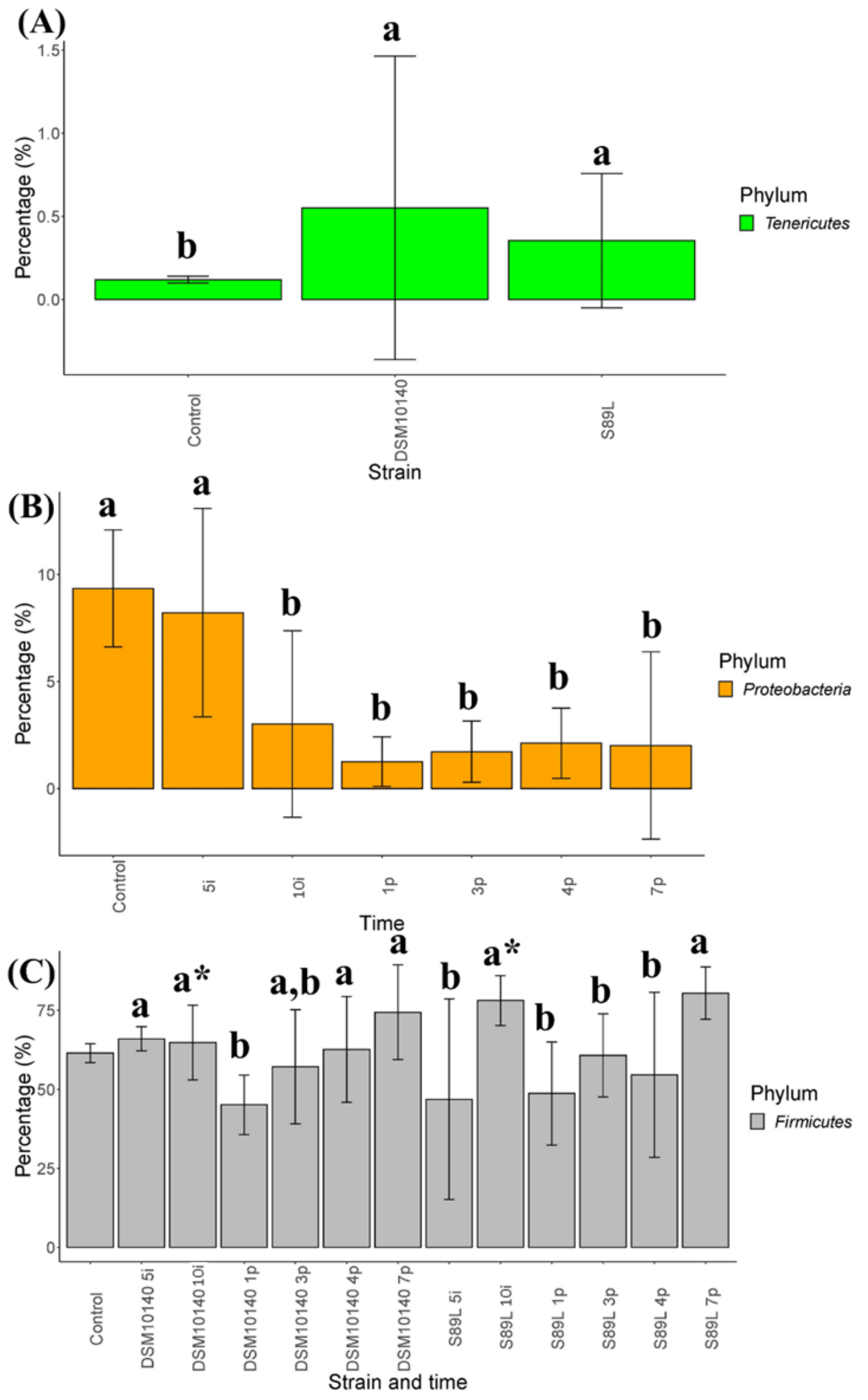

FIGURE S3 | Statistically significant differences at phylum level determined by DESeq2 differential abundance testing according to the EPS-strain selected (**A**), the sampling (days) points (**B**) and both EPS-strain and sampling points (**C**). Different letters indicate statistical differences among the different EPS-strain groups (A) or sampling days (B); in the combination of both factors (C), within each EPS-strain group, the samples (days) that do not share a common letter are statistically different ($p<0.05$), whereas within each treatment day, the statistical differences between the DSM10140 and S89L groups are marked with an asterisk (* $p<0.05$).

1Oi

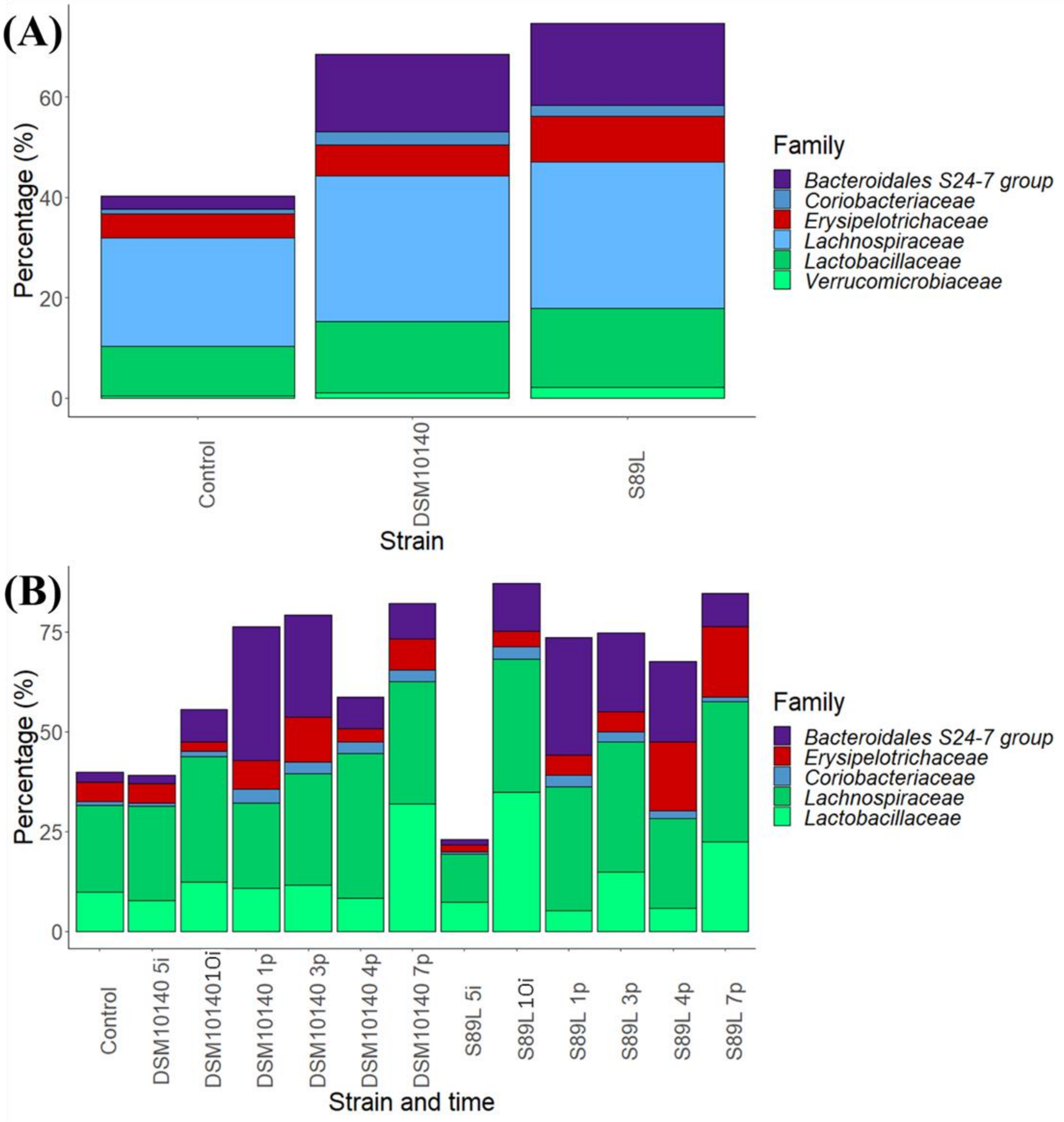

FIGURE S4 | Statistically significant differences at family level determined by DESeq2 differential abundance testing according to the EPS-strain selected (**A**) and both EPS-strain and intervention time (**B**).

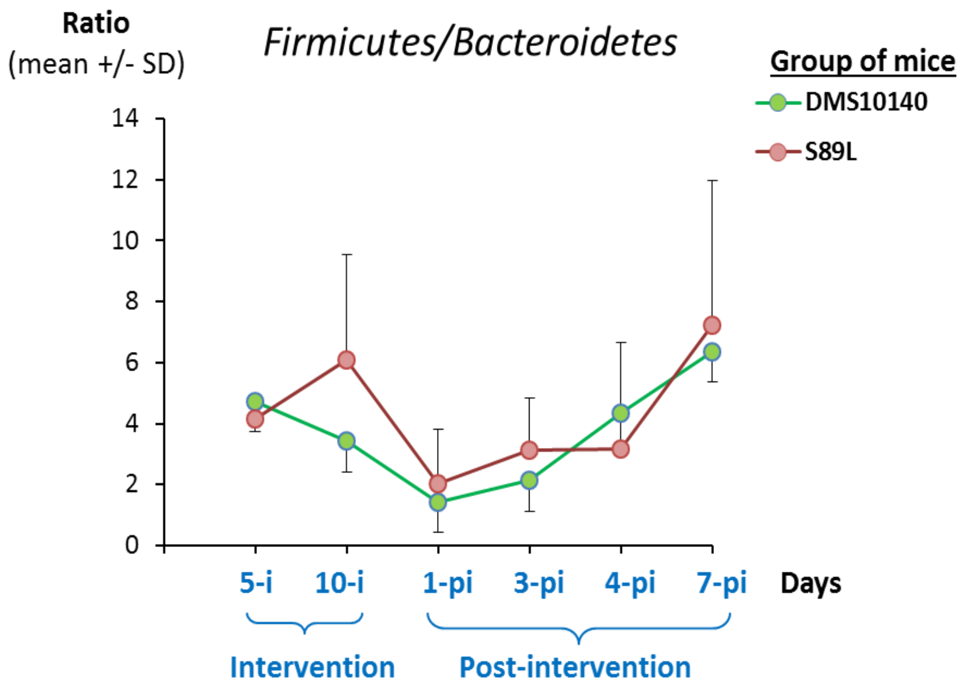

FIGURE S5 | Ratio of the relative abundance of phyla *Firmicutes* and *Bacteroidetes* calculated for the two mice groups at different sampling points along the experimental procedure (See **Figure 3** for detailed explanation).

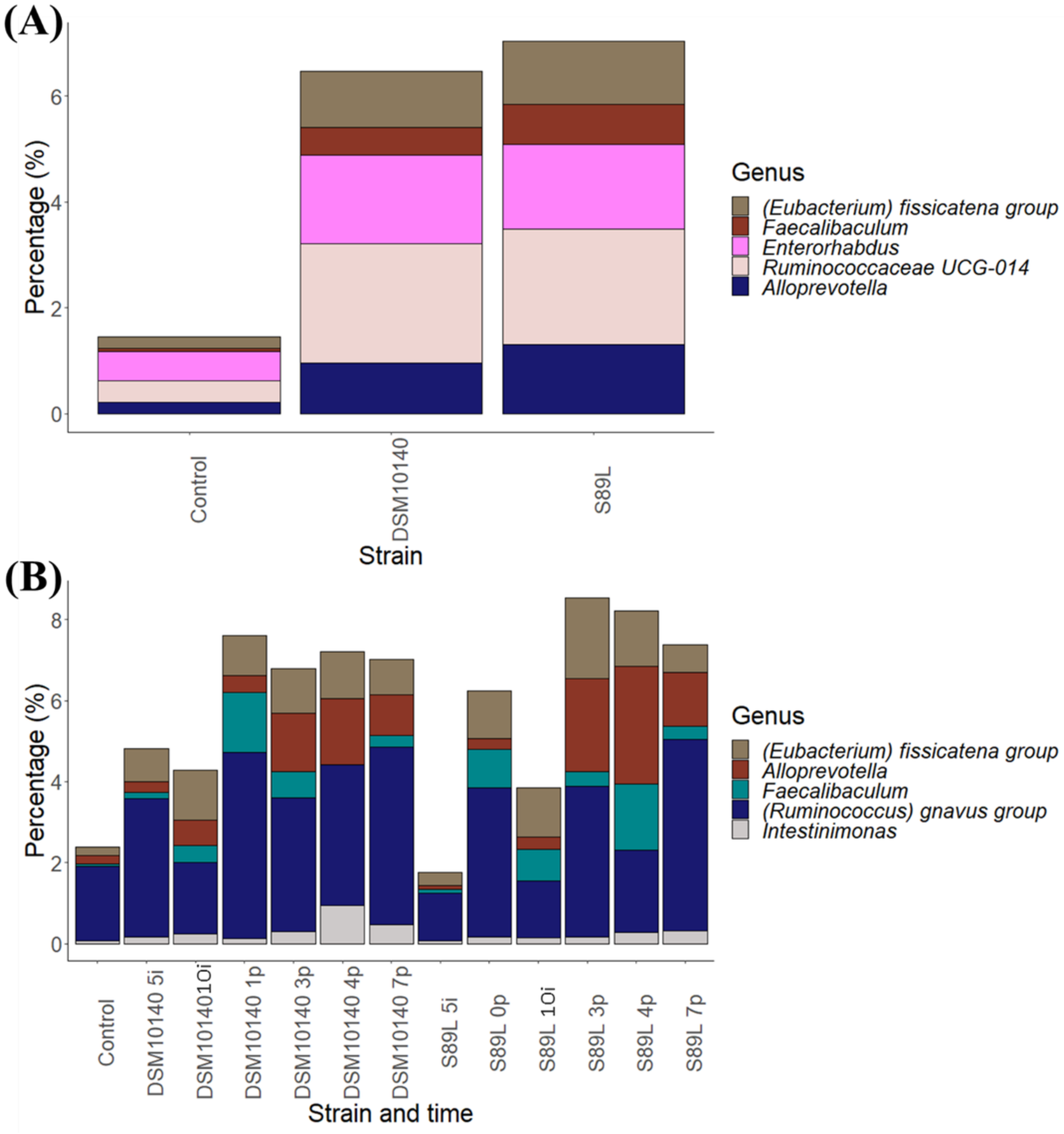

FIGURE S6 | Statistically significant differences at genus level determined by DESeq2 differential abundance testing according to the EPS-strain selected (**A**) and both EPS-strain and intervention time (**B**).

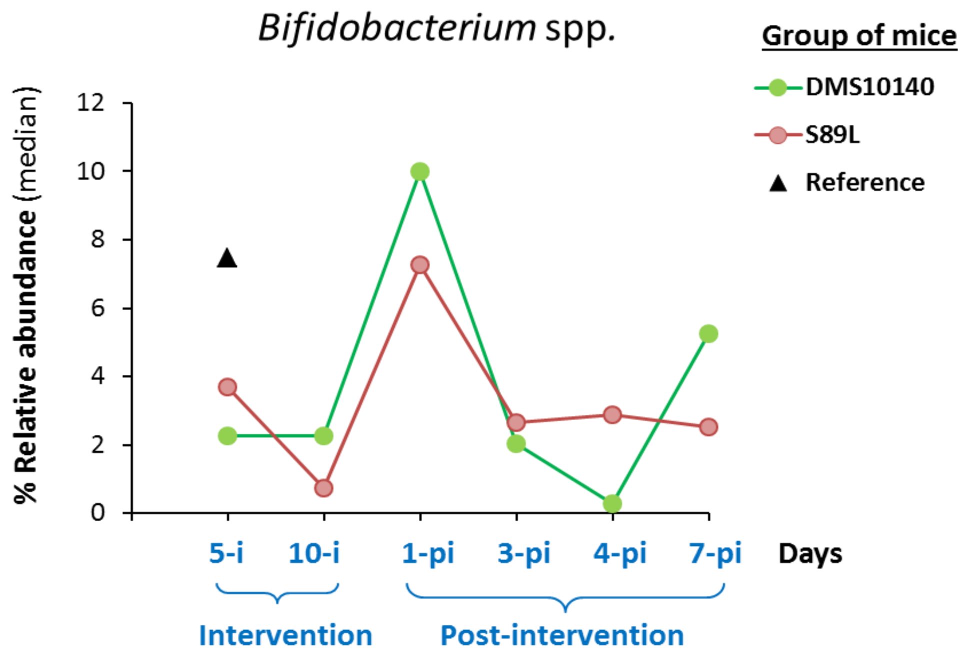

FIGURE S7 | Evolution of the relative abundance (median values of at least 6 mice per point) of *Bifidobacterium* genus in the two mice groups at different sampling points along the experimental procedure (See **Figure 3** for detailed explanation). The abundance of the reference (non-treated) mice group is represented with a triangle.

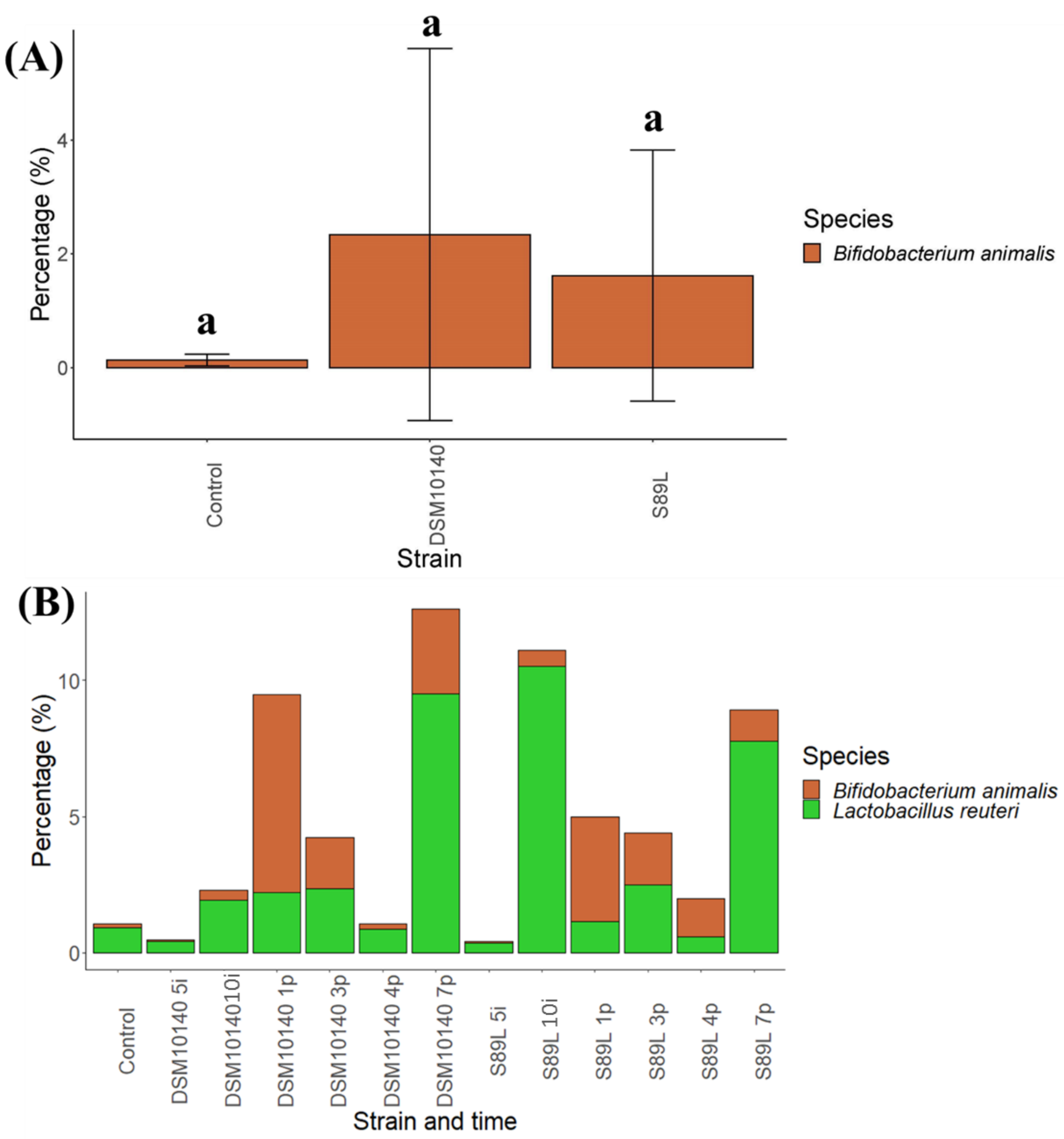

FIGURE S8 | Statistically significant differences at species level determined by DESeq2 differential abundance testing according to the EPS-strain selected (**A**) and both EPS-strain and intervention time (**B**). Different letters indicate statistical differences between samples.

TABLE S1 | Mean and standard deviation of the cytokines quantified (pg/mL) in serum samples collected at different point from BALB/c mice feed with the exopolysaccharide (EPS)-producing strains *Bifidobacterium animalis* subsp. *lactis* DSM10140 (parental, non-ropy strain) or S89L (mutant, ropy strain producing a high molecular weight EPS). Within each strain-treatment group and for each cytokine, the non-parametric Kruskal-Wallis test for K-independent samples (p-value) was used to assess differences among the treatment days; afterwards, the Mann-Whitney test for 2-independent samples was used to assess differences between each pair of days and, in this case, those that do not share a common letter within each row are statistically different ($p<0.05$). Within each treatment day and for each cytokine, the statistical differences between the DSM10140 and S89L groups are marked with an asterisk (*$p<0.05$), as analyses by means of Mann-Whitney test for 2-independent samples. The basal values of the reference (control) group were: 2.38±0.52 (IL-5), 5.25±0.01 (IL-6), 33.24±43.43 (IL-9), 52.61±49.15 (IL-10), 1.23±0.01 (IL-17a), 12.72±0.01 (IL-23), 18.72±15.27 (IL-27), 43.39±78.65 (IFN-γ), 3.71±4.89 (IL-10/ IFN-γ), and 42.67±39.86 (IL-10/IL-17a).

		**Intervention period**		**Post-intervention** (without bifidobacteria administration)				
**Cytokine**	**Strain**	**5 days**	**10 days**	**1 day**	**3 days**	**4 days**	**7 days**	**p-value**
**IL-5**	DSM10140	5.95±5.70	3.39±2.33	3.72±2.95	3.51±2.01	3.99±2.51	10.59±17.25	0.907
	S89L	2.78±2.16	6.81±9.17	6.32±6.82	5.48±3.57	3.85±2.82	5.39±3.35	0.808
**IL-6**	DSM10140	4.78±0.94 [a]	75.44±198.52 [ab]	14.69±21.39 [ab]	5.32±0.37 [a]	44.98±60.89 [b]	6.48±2.09 [a]	**0.022**
	S89L	13.82±16.96	7.28±4.07	6.28±5.74	7.44±4.73	14.91±25.23	4.40±1.50	0.502
**IL-9**	DSM10140	44.97±27.79	40.56±32.19	52.02±31.71	57.97±53.98	149.05±247.86	245.17±362.71	0.838
	S89L	495.7±426.5 *	368.55±378.53	648.16±675.84 *	540.36±319.76 *	385.83±181.73	211.15±237.52	0.295
**IL-10**	DSM10140	41.61±63.55	43.77±53.47	19.34±19.73	36.81±56.79	29.05±37.96	84.01±83.05	0.736
	S89L	224.5±145.8	185.58±183.71	109.48±175.19	180.26±133.42 *	155.75±224.69	65.30±93.21	0.326
**IL-17a**	DSM10140	1.23±0.01	1.23±0.01	1.49±0.82	1.23±0.01	2.60±3.05	3.20±3.13	0.197
	S89L	4.48±0.67 *	4.09±3.50 *	5.51±5.32 *	4.79±2.94 *	5.53±4.60	3.31±3.78	0.819

TABLE S1 | Continuation

Cytokine	Strain	Intervention period		Post-intervention (without bifidobacteria administration)				p-value
		5 days	10 days	1 day	3 days	4 days	7 days	
**IL-23**	DSM10140	12.72±0.01	12.72±0.01	11.21±4.27	12.72±0.01	26.67±30.60	32.39±28.50	0.180
	S89L	53.13±19.44 *	36.34±18.74 *	47.19±37.49 *	47.92±50.35 *	50.10±73.96	36.21±17.75	0.589
**IL-27**	DSM10140	33.02±12.01	37.69±54.99	469.59±1243.47	28.75±31.02	194.58±304.52	422.11±471.39	0.670
	S89L	231.1±281.1	622.51±475.43 *	802.76±627.26 *	544.89±409.78 *	595.01±415.86	449.60±693.04	0.288
**IFN-γ**	DSM10140	3.00±1.14 [a]	3.88±1.23 [a]	2.60±1.73 [a]	31.32±73.67 [ab]	68.90±105.83 [b]	9.60±8.00 [ab]	**0.033**
	S89L	35.24±40.77 *	10.27±5.01 *	15.07±4.07 *	25.78±23.70 *	17.28±14.30	11.93±4.04	0.085
**Ratio IL-10/ IFN-γ**	DSM10140	11.76±16.87	11.97±14.45	19.13±31.90	15.36±21.23	1.26±1.69	9.79±10.90	0.215
	S89L	14.60±10.89	17.29±18.14	8.72±15.61	9.24±7.05	8.48±12.56	4.82±7.32	0.346
**Ratio IL-10 /IL17a**	DSM10140	33.74±51.54	35.50±43.39	15.59±16.11	29.86±46.06	15.21±22.98	34.80±37.28	0.840
	S89L	50.75±31.13	111.28±149.21	37.11±79.34	45.79±35.67	27.51±32.17	31.85±53.93	0.550

TABLE S2 | Mean and standard deviation of the relative abundance of the main (≥ 1%) bacterial phyla detected in the colon content of different BALB/c mice groups (n≥ 6 per group in sampling day) treated with the exopolysaccharide (EPS)-producing strains *Bifidobacterium animalis* subsp. *lactis* DSM10140 (parental, non-ropy strain) or S89L (mutant, ropy strain producing a high molecular weight EPS). Within each strain-treatment group and for each phylum, the non-parametric Kruskal-Wallis test for K-independent samples (p-value) was used to assess differences among the treatment days; afterwards, the Mann-Whitney test for 2-independent samples was used to assess differences between each pair of days and, in this case, those that do not share a common letter within each row are statistically different ($p<0.05$). Within each treatment day and for each phylum, the statistical differences between the DSM10140 and S89L groups are marked with an asterisk (*$p<0.05$), as analyses by means of Mann-Whitney test for 2-independent samples.

		**Intervention period**		**Post-intervention** (without bifidobacteria administration)				
**Phylum**	**Strain**	**5 days**	**10 days**	**1 day**	**3 days**	**4 days**	**7 days**	**p-value**
Actinobacteria	DSM10140	6.48 ± 2.34 ^{a}	6.17 ± 2.19 ^{a}	12.66 ± 3.89 ^{b}	7.15 ± 4.36 ^{ab}	4.25 ± 1.63 ^{a}	7.32 ± 3.3 ^{ab}	0.033
	S89L	9.25 ± 4.08 ^{b}	4.57 ± 1.51 ^{a}	10.48 ± 4.95 ^{b}	5.56 ± 2.45 ^{ab}	5.85 ± 1.79 ^{ab}	3.77 ± 2.56 ^{a}	0.041
Bacteroidetes	DSM10140	15.05 ± 2.72 ^{ab}	19.36 ± 9.46 ^{ab}	35.87 ± 6.57 ^{c}	31.2 ± 19.45 ^{bc}	19.78 ± 12.39 ^{ab}	12.01 ± 9.38 ^{a}	0.013
	S89L	15.67 ± 1.51 ^{a}	13.73 ± 5.81 ^{a}	32.48 ± 15.84 ^{b}	25.39 ± 10.92 ^{ab}	30.54 ± 23.94 ^{ab}	13.20 ± 6.27 ^{a}	0.048
Firmicutes	DSM10140	67.64 ± 4.3 ^{b}	64.93 ± 11.66 ^{b}	47.09 ± 7.23 ^{a}	57.02 ± 16.85 ^{ab}	68.6 ± 12.75 ^{b}	74.3 ± 14.02 ^{b}	0.009
	S89L	64.31 ± 6.63 ^{a}	77.88 ± 7.78 ^{b}	46.92 ± 15.10 ^{a}	63.77 ± 10.14 ^{a}	56.81 ± 25.30 ^{ab}	81.11 ± 7.97 ^{b}	0.002
Proteobacteria	DSM10140	8.06 ± 2.77 ^{c}	5.06 ± 4.98 ^{abc}	1.59 ± 1.38 ^{a}	1.77 ± 1.1 ^{ab}	3.38 ± 1.63 ^{b}	3.47 ± 5.64 ^{ab}	0.011
	S89L	7.14 ± 4.94 ^{b}	0.77 ± 0.75 ^{a}	1.26 ± 0.77 ^{a}	1.66 ± 1.74 ^{a}	0.83 ± 0.62 ^{a}	0.63 ± 0.54 ^{a}	0.018
			*			*	*	
Saccharibacteria	DSM10140	0.68 ± 0.45	1.19 ± 0.68	1.84 ± 1.53	2.02 ± 2.38	1.7 ± 1.05	1.47 ± 0.89	0.526
	S89L	0.69 ± 0.38 ^{ab}	2.62 ± 1.57 ^{c}	1.74 ± 1.09 ^{bc}	2.14 ± 2.00 ^{bc}	0.32 ± 0.30 ^{a}	0.23 ± 0.23 ^{a}	0.000
						*	*	
Tenericutes	DSM10140	1.06 ± 1.64 ^{ab}	1.42 ± 0.9 ^{b}	0.22 ± 0.10 ^{ab}	0.28 ± 0.39 ^{a}	0.38 ± 0.51 ^{ab}	0.23 ± 0.16 ^{a}	0.048
	S89L	1.50 ± 2.02 ^{c}	0.24 ± 0.18 ^{ab}	0.44 ± 0.30 ^{bc}	0.67 ± 0.48 ^{bc}	0.40 ± 0.51 ^{abc}	0.14 ± 0.13 ^{a}	0.057
			*					
Verrucomicrobia	DSM10140	0.36 ± 0.06	1.27 ± 1.97	0.56 ± 0.81	0.38 ± 0.8	1.71 ± 2.74	1.06 ± 2.16	0.171
	S89L	0.31 ± 0.18 ^{bc}	0.06 ± 0.09 ^{a}	6.50 ± 9.91 ^{abc}	0.55 ± 1.11 ^{ab}	5.11 ± 3.68 ^{c}	0.81 ± 0.83 ^{abc}	0.038
			*					

TABLE S3 | Mean and standard of the relative abundance (≥ 1%) of the bacterial families showing statistical differences between the two BALB/c mice groups (n≥ 6 per group in sampling day) within each treatment day: treated with the exopolysaccharide (EPS)-producing strains *Bifidobacterium animalis* subsp. *lactis* DSM10140 (parental, non-ropy strain) or S89L (mutant, ropy strain producing a high molecular weight EPS). The Metastats statistical method (non-parametric for 2 non-related samples), with a FDR (false discovery rate) adjusted with Benjamini-Hochberg method to of 0.25, was used to assess the statistical differences between the two treatment groups. The software package Mothur vs1.39.5 was used for this analysis.

		**Mean ± SD (n≥6)**			
**Treatment period**	**Family**	**DSM10140 group**	**S89L group**	***p*-value**	**Fold-change*** (in strain S89L)
**10 days of intervention**	*Lactobacillaceae*	8.38 ± 5.17	37.85 ± 13.78	0.000033	4.5 (↑)
	Rikenellaceae	2.47 ± 0.67	0.96 ± 0.54	0.000039	2.6 (↓)
	Bifidobacteriaceae	2.29 ± 0.42	1.12 ± 0.82	0.000824	2.0 (↓)
	Coriobacteriaceae	1.57 ± 0.89	3.36 ± 1.20	0.001235	2.1 (↑)
	Porphyromonadaceae	1.71 ± 1.18	0.32 ± 0.17	0.001464	5.3 (↓)
	Ruminococcaceae	11.83 ± 7.33	4.45 ± 2.44	0.007209	2.7 (↓)
	Bacteroidaceae	2.93 ± 2.98	0.31 ± 0.55	0.015275	9.5 (↓)
**4 days post-intervention**	*Bifidobacteriaceae*	0.55 ± 0.46	3.70 ± 1.85	0.000319	6.7 (↑)
	Desulfovibrionaceae	1.01 ± 0.53	0.23 ± 0.27	0.002416	4.4 (↓)
**7 days post-intervention**	*Coriobacteriaceae*	3.00 ± 1.07	1.06 ± 0.52	0.000282	2.8 (↓)

***** This value was calculated dividing the biggest number by the smallest number; the arrow indicates the change of relative abundance in the group fed with the strain S89L with respect to the group feed with the strain DSM10140.
